# Theory of anisotropic superfluid ^4^He counterflow turbulence

**DOI:** 10.1098/rsta.2021.0094

**Published:** 2022-03-21

**Authors:** Victor S. L'vov, Yuri V. Lvov, Sergey Nazarenko, Anna Pomyalov

**Affiliations:** ^1^ Department of Chemical and Biological Physics, Weizmann Institute of Science, Rehovot, Israel; ^2^ Rensselaer Polytechnic Institute, Troy, NY 12180, USA; ^3^ Institut de Physique de Nice, Université Cote d’Azur, CNRS, Nice, France

**Keywords:** liquid ^4^He, superfluid turbulence, anisotropic energy spectra, thermal counterflow

## Abstract

We develop a theory of strong anisotropy of the energy spectra in the thermally driven turbulent counterflow of superfluid ^4^He. The key ingredients of the theory are the three-dimensional differential closure for the vector of the energy flux and the anisotropy of the mutual friction force. We suggest an approximate analytic solution of the resulting energy-rate equation, which is fully supported by our numerical solution. The two-dimensional energy spectrum is strongly confined in the direction of the counterflow velocity. In agreement with the experiments, the energy spectra in the direction orthogonal to the counterflow exhibit two scaling ranges: a near-classical non-universal cascade dominated range and a universal critical regime at large wavenumbers. The theory predicts the dependence of various details of the spectra and the transition to the universal critical regime on the flow parameters.

This article is part of the theme issue ‘Scaling the turbulence edifice (part 2)’.

## 1. Introduction

Most universal properties of turbulence are only revealed in flows with very high Reynolds number. Typically, such conditions are found in atmospheric turbulence or in very large wind tunnels. Liquid helium has very low kinematic viscosity and, therefore, becomes an ideal test-bed for high-Reynolds number turbulence even in a relatively small experimental facility. The liquid helium viscosity decreases with temperature and below the Bose–Einstein condensation temperature $T_{\lambda }\approx 2.17\;\text{K}$ ^4^He becomes superfluid. In this state, it can be described as a two-component fluid in which a viscous normal-fluid and an inviscid superfluid components interact via a mutual friction force [1–10].

Various ways of turbulence generation in superfluid He produce flows with very different properties. Mechanically driven superfluid He with two components flowing in the same direction and coupled by the mutual friction almost at all scales has long been considered similar [7–9] to the classical flows [11]. The similarity included the behaviour of the structure functions and scaling of the turbulent energy spectra close to $k^{-5/3}$ [9,12–18].

The two-fluid nature of the superfluid ^4^He allows generation of turbulence by thermal gradient [2,5,8,19–22]. In such a flow that has no classical analogy, the two fluid components flow in opposite directions: the normal fluid carries the heat flux away from the heat source with the mean velocity $U_{n}$, while the superfluid flows towards the heater with the mean velocity $U_{s}$. The mutual friction force that couples the components leads to both the energy exchange and additional dissipation by mutual friction that are scale-dependent [23,24]. Since all relevant fluid parameters [25] are strongly temperature-dependent, the statistical properties of such a counterflow are not universal. Instead, the statistics of the counterflow depends on the temperature and on the relative velocity $U_{ns}=U_{n}-U_{s}$ [24,26–30]. Recent flow visualization experiments [27,28,31–33] stimulated theoretical and numerical investigations of the energy spectra of the counterflow turbulence. It was shown [23,24,30,34,35] that besides the dependence on flow parameters, the energy spectra are sensitive to the angle with respect to the direction of the counterflow velocity. As a result, the energy spectra in the counterflow turbulence are anisotropic and strongly suppressed in the direction of $U_{ns}$.

Although such a spectral anisotropy was predicted theoretically and confirmed numerically [29,30], the experimental investigations of the energy spectra for the time being are limited to the plane, orthogonal to the direction of the counterflow velocity [28,33], while the theory of counterflow turbulence [24] was developed assuming spectral isotropy. In this paper, we relax this assumption and offer a theoretical description of the spectral anisotropy of the energy spectra of the counterflow turbulence in superfluid ^4^He.

The paper is organized as follows. In §2, we develop the theory of anisotropic turbulence. Similar to our previous studies of superfluid turbulence [14,23,24,36,37], we describe the large-scale turbulence in superfluid ^4^He by the coarse-grained Navier–Stokes equation (2.1) coupled by the mutual friction force. These equations are detailed in §2a. In §2b, we introduce various statistical characteristics of anisotropic turbulence, used in our paper. In the focal §2c, we suggest the energy rate equation (2.10) for the axially symmetric counterflow turbulence. The key element [§2(c,ii)] in the resulting energy rate equation (2.10) is the coupling function $D(k_{||})$, which depends only on $k_{||}$, according to equation (2.6b). In §1(c,iii), we introduce a vector energy flux $\varepsilon (k)=\{\varepsilon_{||}(k),\varepsilon_{⊥}(k)\}$, which depends on the position in the plane $k=\{k_{||},k_{⊥}\}$, formed by the components $k_{||}$ and $k_{⊥}$ of the wavevector k, parallel and orthogonal to the counterflow velocity $U_{ns}$, respectively. We analyse the resulting energy rate equation analytically in §3 and numerically in §4. Finally, in §5, we summarize our findings.

## 2. A theory of anisotropic counterflow turbulence

The superfluid phase of liquid He is characterized by quantized vorticity that is constrained to vortex-line singularities of core radius $a_{0}\approx 10^{-8}\;\text{cm}$ and fixed circulation κ=h/M, where h is Planck’s constant and M is the mass of the ^4^He atom [3]. The superfluid turbulence is manifested as a complex tangle of these vortex lines with a typical inter-vortex distance [5] $\ell ∼10^{-4}-10^{-2}\;\text{cm}$.

Large-scale hydrodynamics of such a system is usually described by a two-fluid model, interpreting ^4^He as a mixture of two coupled fluid components: an inviscid superfluid and a viscous normal fluid. The temperature-dependent densities of the normal-fluid and superfluid components $\rho_{s},\rho_{n}:\rho_{s}+\rho_{n}=\rho$ define their contributions to the mixture. Here, ρ is the density of ^4^He. The fluid components are coupled by the mutual friction force, mediated by the tangle of quantum vortexes [1,5,6,9,19–22].

### (a) Coarse-grained equations for counterflow He-4 turbulence

Similar to [30], our approach to the problems of counterflow turbulence with the scales much larger than the intervortex distance [23,24,36] is based on the coarse-grained equations [14,24,36,37] of the incompressible superfluid turbulence. These equations, often called Hall–Vinen–Bekarevich–Khalatnikov equations (HVBK) [38,39], have a form of two Navier–Stokes equations (NSE) for the turbulent velocity fluctuations of the normal fluid and superfluid components $u_{n}(r,t)$ and $u_{s}(r,t)$ in the presence of space-homogeneous mean normal and superfluid velocities $U_{n}$ and $U_{s}$

2.1*a* $$\frac{\partial u_{s}}{\partial t}+[(u_{s}+U_{s})\cdot \nabla ]u_{s}-\frac{1}{\rho_{s}}\nabla p_{s}=\nu_{s}\Delta u_{s}+f_{ns},\;f_{ns}≃\Omega_{s}(u_{n}-u_{s})$$

and

2.1*b* $$\frac{\partial u_{n}}{\partial t}+[(u_{n}+U_{n})\cdot \nabla ]u_{n}-\frac{1}{\rho_{n}}\nabla p_{n}=\nu_{n}\Delta u_{n}-\frac{\rho_{s}}{\rho_{n}}f_{ns},\;\Omega_{s}=\alpha (T)\kappa L,$$

coupled by the mutual friction force $f_{ns}$ in the form (2.1a) and complemented by the incompressibility conditions

2.1*c* $$\nabla \cdot u_{n}=0\;\text{and}\;\nabla \cdot u_{n}=0.$$

The mutual friction force involves the temperature dependent dimensionless dissipative mutual friction parameter α(T) and the superfluid vorticity κL. Here, L is the vortex line density (VLD). Furthermore, the partial densities of the normal and superfluid components are $\rho_{n}$ and $\rho_{s}$, the pressure of the normal and the superfluid components are

2.1*d* $$p_{n}=\frac{\rho_{n}}{\rho }\left[p+\frac{\rho_{s}}{2}|U_{ns}+u_{n}-u_{s}{|}^{2}\right]\;\text{and}\;p_{s}=\frac{\rho_{s}}{\rho }\left[p-\frac{\rho_{n}}{2}|U_{ns}+u_{n}-u_{s}{|}^{2}\right].$$

The kinematic viscosity of normal fluid component $\nu_{n}=\eta /\rho_{n}$ with η being the dynamical viscosity [25] of normal ^4^He component and the Vinen’s effective superfluid viscosity [5] $\nu_{s}$, which accounts [36] for the energy dissipation at the intervortex scale ℓ due to vortex reconnections, the energy transfer to Kelvin waves and other dissipation mechanisms.

We consider here the planar heat source, typically used in the channel counterflow.

### (b) Statistical characteristics of anisotropic turbulence

The general description of the homogeneous superfluid ^4^He turbulence at the level of the second-order statistics can be done in terms of the three-dimensional correlation functions of the normal-fluid and superfluid turbulent velocity fluctuations in the k-representation

2.2 $${\left(2\pi \right)}^{3}\delta^{3}(k-k^{\prime })F_{ij}^{\alpha \beta }(k)=\left\langle v_{i}^{\alpha }(k)\cdot v_{j}^{*\beta }(k^{\prime })\right\rangle ,\;F_{ij}(k)\equiv \sum_{\alpha =x,y,z}F_{ij}^{\alpha \alpha }(k).$$

Here, $F_{j}^{\alpha \beta }(k)=F_{jj}^{\alpha \beta }(k)$, $\delta^{3}(k-k^{\prime })$ is three-dimensional Dirac’s delta function and

2.3 $$v_{j}(k)=\int u_{j}(r)\exp(ik\cdot r)\text{d}r\;\text{and}\;u_{j}(r)=\int v_{j}(k)\exp(-ik\cdot r)\frac{\text{d}k}{{\left(2\pi \right)}^{3}}.$$

The subscripts ${\;}_{i,j}$ in Eq (2.2) denote the normal (i,j=n) or the superfluid (i,j=s) fluid components and ${\;}^{*}$ stands for complex conjugation. The three-dimensional correlation function $F_{ij}(k)$ and the Fourier transform (2.3) are defined such that the kinetic energy density per unit mass $E_{j}$ (with the dimension $[E]={\text{cm}}^{2}\;{\text{s}}^{-2}$) reads

$$E_{j}=\frac{1}{2}\left\langle |u_{j}(r){|}^{2}\right\rangle =\frac{1}{2}\int F_{jj}(k)\frac{d^{3}k}{{\left(2\pi \right)}^{3}}.$$

Due to the presence of the preferred direction, defined by the counterflow velocity $U_{ns}$, the counterflow turbulence has an axial symmetry around that direction. In this case, $F_{ij}^{\alpha \beta }(k)$ depends only on two projections $k_{||}$ and $k_{⊥}$ of the wave-vector k: $k_{||}\equiv U_{ns}(k\cdot U_{ns})/U_{ns}^{2}$ and $k_{⊥}=(k-k_{||})$, being independent of the angle φ in the ⊥-plane, orthogonal to $U_{ns}$: $E_{ij}^{\alpha \beta }(k)\Rightarrow E_{ij}^{\alpha \beta }(k_{||},k_{⊥})$.

In the case of axial symmetry, a two-dimensional object $E_{ij}^{\alpha \beta }(k_{||},k_{⊥})$ still contains all the information about second-order statistics of the counterflow turbulence: $E_{j}(k_{||},k_{⊥})\equiv (k_{⊥}/4\pi^{2})F_{j}(k_{||},k_{⊥})$. Now the total kinetic energy density per unit mass can be found as $E_{j}=\int \int_{0}^{\infty }\;\text{d}k_{||}\;\text{d}k_{⊥}E_{j}(k_{||},k_{⊥})$. In the fully isotropic case, $E_{j}(k_{||},k_{⊥})$ depends only on $k=\sqrt{k_{||}^{2}+k_{⊥}^{2}}$ and we can introduce traditional one-dimensional energy spectrum

2.4 $${\tilde{E}}_{j}(k)=2\pi kE_{j}(k_{||},k_{⊥}).$$

### (c) Energy rate equations for counterflow turbulence

#### (i) General form of the energy rate equation in axial symmetry

A theory of space-homogeneous counterflow turbulence [24], developed under simplifying assumption of the spectral isotropy of the flow, is based on the stationary balance equations for the one-dimensional energy spectra ${\tilde{E}}_{j}(k)$, (2.4). Here, we relax the assumption of the isotropy, and derive an energy rate equation for the two-dimensional energy spectra $E_{j}(k_{||},k_{⊥})$ of the counterflow turbulence with axial symmetry around $k_{||}$. To this end, we, following [24], eliminate the pressure terms using the incompressibility conditions, Fourier transform and multiply them by the complex conjugates of the corresponding velocities. After ensemble averaging, we get the equations for the three-dimensional spectra $F_{j}(k)$, defined by (2.2), and average them only over the azimuth angle φ in the plane orthogonal to $k_{||}$. Finally, we get

2.5 $$\frac{\partial E_{j}(k,t)}{\partial t}+{\text{div}}_{k}[\varepsilon_{j}(k)]=\Omega_{j}[E_{ns}(k)-E_{j}(k)]-2\nu_{j}k^{2}E_{j}(k),\;\Omega_{n}=\frac{\Omega_{s}\rho_{s}}{\rho_{n}}.$$

Here, $k=\{k_{||},k_{⊥}\}$ is a two-dimensional wavevector, $\varepsilon_{j}(k)=\{\varepsilon_{j}^{||},(k),\varepsilon_{j}^{⊥}(k)\}$ is the vector of the energy flux. The cross-correlation function $E_{ns}$ is discussed in the next section and the derivation of the vector energy flux is detailed in §2c(iii).

#### (ii) Cross-correlation function in counterflow turbulence

In our analysis, we use the model of the anisotropic cross-correlation function $E_{ns}(k_{||},k_{⊥})$, introduced by equation (13) of [23]:

2.6*a* $$E_{ns}(k)=\frac{A(k)\Omega_{ns}}{\Omega_{ns}^{2}+{\left(k_{||}U_{ns}\right)}^{2}},\;A(k)=\Omega_{s}E_{n}(k)+\Omega_{n}E_{s}(k),\;\Omega_{ns}=\Omega_{n}+\Omega_{s}.$$

Further simplifications [24] allow one to rewrite (2.6a) for $E_{ns}(k)$ in the following form:

2.6*b* $$E_{ns}(k)=E_{j}(k)D(k_{||}),\;D(k_{||})=\frac{k_{\times }^{2}}{(k_{\times }^{2}+k_{||}^{2})},\;k_{\times }=\frac{\Omega_{ns}}{U_{ns}}.$$

Note that while substituting $E_{ns}(k)$ into the rate (2.5), we should take in (2.6b) j=n in the equation for the normal component, and j=s for the superfluid component.

The physical meaning of the two-dimensional coupling function $D(k_{||})$ in (2.6b) is the same as in the spherical case: it describes the level of decorrelation of the normal-fluid and superfluid velocity components by the counterflow velocity. For $k_{||}≲k_{\times }$, normal-fluid and superfluid velocities are almost fully coupled. In this case, the mutual friction only in Eq (2.2) weakly affects the energy balance. The energy spectrum in the inertial interval of scales is determined by the step-by-step cascade energy transfer. Accordingly, this range of wavenumbers can be called ‘cascade-dominated’ [24]. For large $k_{||}$, $D(k_{||})\ll 1$ and the velocities of fluid components are almost decoupled. In this ‘mutual-friction dominated range’, the energy dissipation by mutual friction strongly suppresses the energy spectra.

#### (iii) The energy transfer term

The energy transfer term ${\text{div}}_{k}[\varepsilon_{j}(k)]$ in (2.5) originates from the nonlinear terms in the coupled NSE equation (2.1) and has the same form [40–42] as in the classical turbulence

2.7 $$\begin{array}{rl} & {\text{div}}_{k}[\varepsilon_{j}(k)]\equiv \frac{\text{d}\varepsilon_{j}(k)}{\text{d}k}=2\;\text{Re}\{\int V^{\xi \beta \gamma }(k,q,p)E_{j}^{\xi \beta \gamma }(k,q,p)\delta (k+q+p)\frac{d^{3}q\;d^{3}p}{{\left(2\pi \right)}^{6}}\}\\ \;\text{and}\; & V^{\xi \beta \gamma }(k,q,p)=i(\delta_{\xi \xi^{\prime }}-\frac{k^{\xi }k^{\xi^{\prime }}}{k^{2}})(k^{\beta }\delta_{\xi^{\prime }\gamma }+k^{\gamma }\delta_{\xi^{\prime }\beta }). \end{array}\}$$

Here, $E_{j}^{\xi \beta \gamma }(k,q,p)$ is the simultaneous triple-correlation function of turbulent (normal or superfluid) velocity fluctuations in the k-representation, that we will not specify here and $V^{\xi \beta \gamma }(k,q,p)$ is the interaction vertex in the NSE. Importantly, the right-hand side of (2.7) conserves the total turbulent kinetic energy (i.e. the integral of $E_{j}(k)$ over entire k-space) and therefore can be written in the divergent form as ${\text{div}}_{k}[\varepsilon_{j}(k)]$.

A simple algebraic closure approximation for the energy flux $\tilde{\varepsilon }(k)$ in isotropic turbulence follows from the dimensional reasoning in the framework of the Kolmogorov 1941 (K41) hypothesis [11]

2.8*a* $$\tilde{\varepsilon }(k)=\tilde{C}k^{5/2}{\tilde{E}}^{3/2}(k).$$

Here, $\tilde{C}$ is a dimensionless constant of the order of unity and $\tilde{\varepsilon }$ is the energy flux in the inertial interval of scales. Equation (2.8a) immediately gives the celebrated $\frac{5}{3}$-law: ${\tilde{E}}_{K41}(k)=C_{K41}{\tilde{\Omega }}^{2/3}k^{-5/3}$ with $C_{K41}={\tilde{C}}^{-2/3}$. The experimental value [43] of the constant $C_{K41}≃0.5$. In the two-dimensional case with axial symmetry along the counterflow direction, the situation is more involved. Now, the two-dimensional vector ε with the dimensions $[\varepsilon ]={\left(\text{cm}\;{\text{s}}^{-1}\right)}^{3}$ is the flux of two-dimensional-energy density E(k) per unit mass per square of unit k with the dimensions $[E]={\text{cm}}^{4}\;{\text{s}}^{-2}$. The dimensional reasoning, similar to that leading to (2.8a) gives

2.8*b* $$|\varepsilon (k)|\approx Ck^{3}E_{j}^{3/2}(k),\;k=\{k_{||},k_{⊥}\},$$

with $C=\tilde{C}/\sqrt{2\pi }≃1.1$.

Unfortunately, the dimensional reasoning does not allow us to reconstruct the direction of the vector ε. It is natural to assume that ε is oriented in the direction of the steepest descent of the three-dimensional energy spectrum, i.e. along $\nabla_{k}[E(k)/k]$ or, if this gradient is zero, ε=0. Note that this allows us to satisfy an additional physical requirement that the energy flux vanishes in the thermodynamic equilibrium with equipartition of energy, when E(k)∝k [44,45]. Thus, requiring the Kolmogorov-type scaling properties, we choose the energy flux in the form $\varepsilon \propto \nabla_{k}{\left[E(k)/k\right]}^{3/2}$. Reconstructing the prefactor according to (2.8b), one finds

2.9*a* $$\varepsilon (k)=-C_{1}k^{11/2}\nabla_{k}[\frac{E(k)}{k}{]}^{3/2},\;\nabla_{k}\equiv \frac{\text{d}}{\text{d}k},$$

with some new dimensionless coefficient $C_{1}\approx 2C/11≃0.2$. The numerical factor is chosen such that closures (2.8b) and (2.9a) coincide for K41 spectrum $E(k)\propto k^{-8/3}$. In the isotropic two-dimensional case, ε(k)∝1/k. This gives $E(k)\propto k^{-8/3}$, as required.

It was shown previously [24,27–30,33] that the energy spectra in the counterflow do not have a simple power-law form in the inertial interval. To account for that it was proposed [24] to replace $C_{1}$ by a function $C_{1}(k)$ that depends on the local slope of the spectrum. Here, we use the same approach and introduce the coefficient

2.9*b* $$C_{1}(k)=\frac{4C_{1}}{3[4-m(k)]},\;m(k)=-k\cdot \nabla_{k}\ln E(k),$$

that depends self-consistently on the local slope m(k) of the energy spectra in the steepest descent direction. The function $C_{1}(k)$ increases when m approaches the critical value m=4, at which the energy transfer over scales looses its locality and, formally, ε→∞.

For m>4, the energy flux in a range from some $\tilde{k}$ to $k\gg \tilde{k}$ becomes non-local (similar to ${\;}^{3}\text{He}$) and requires a more sophisticated closure [37].

#### (iv) Final form of the energy rate equation

Combining equation (2.5) with equations (2.6b), (2.9a) and (2.9b) and neglecting the viscosity term, in the stationary case we finally have

2.10 ∂Ej(k,t)∂t−∇k⋅{C1j(k) k11/2∇k[Ej(k)k]3/2}=−ΩjEj(k) k||2k||2+k×2,k={k||,k⊥}.

Recall that $\Omega_{s}=\alpha \kappa L$, $\Omega_{n}=\Omega_{s}\rho_{s}/\rho_{n}$ and $\Omega_{ns}=\Omega_{s}\rho /\rho_{n}$. The crossed term with time derivative is preserved here (and in some equations below) to stress that this is a continuity equation for the energy spectrum. In theoretical analysis, we will use only the stationary version of this (and similar) equations, while numerically we consider its full version and look for its stationary solutions by numerically integrating continuity equation from appropriate initial conditions.

To simplify the appearance of the energy rate equation (2.10) and to open a way to its numerical solution, we introduce a new function $\Psi_{j}(q,t)$ instead of $E_{j}(k,t)$

2.11 $$E(k)=E(k_{0})\Psi^{2}(q)q^{-8/3},\;q\equiv k/k_{0},$$

such that the fast K41 dependence of E(k) is explicitly accounted for: with K41 scaling Ψ(q)=const. Here, $E(k_{0})$ is the energy spectrum at some $k=k_{0}$ (i.e. for q=1) in the energy containing interval.

Now, equations (2.10) and (2.11) give

2.12 ∂Ψ2∂τ+C(q)q8/3[112q2(q⋅∇q)Ψ3−|∇q|2Ψ3]=−Ω~Ψ2q||2q||2+q×2,∇q≡ddqandC(q)=2C12+3(q⋅∇q)Ψ,Ω~=Ωk03E(k0),τ=tk03E(k0),|∇q|2≡ddq⋅ddq,}

where $q_{\times }=\Omega_{ns}/(k_{0}U_{ns})$ and we neglected the q-derivative of slow function C(q) and took into account that in two-dimensional $\nabla_{q}\cdot (q/q^{2})=0$. Here, for the brevity we skip the index j, keeping in mind that this equation is valid for both the superfluid (with j=s) and for the normal-fluid component (with j=n). After explicit differentiation and division of the resulting equation by Ψ we get

2.13 2∂Ψ2∂τ+3C(q)q8/3[11Ψ2q2(q⋅∇q)Ψ−Ψ|∇q|2Ψ−2|∇qΨ|2]=−Ω~Ψq||2q||2+q×2.

We see that the gradient of function Ψ(q) is present in each term in the square brackets in the left-hand side of (2.13). Therefore, for zero right-hand side (RHS), this equation admits a solution Ψ(q)=const.

The dimensionless parameters $\tilde{\Omega }$ and $q_{\times }$ quantify the mutual friction force. In typical laboratory experiments [28,33], $q_{\times }$ belongs to the interval $q_{\times }\in [1,8]$, while ${\tilde{\Omega }}_{n}\in [3,12]$. In DNS [29,30], $q_{\times }\approx 1.3$, ${\tilde{\Omega }}_{n}≃3$. Having in mind comparison of these results with ours we will analyse (2.13) in the following range of parameters:

2.14 $$q_{\times }\in [1,\;25],\;{\tilde{\Omega }}_{n}\in [2,\;15],\;C_{1}\in [0.1,\;0.5].$$

For T≈1.87 K, we approach the so-called symmetric case with $\rho_{n}\approx \rho_{s}$. Furthermore, we can reasonably assume that both components are equally forced, $E_{n}(k_{0})=E_{s}(k_{0})$. In this case, we can put j=s=n, considering one equation $E(k)=E_{n}(k)=E_{s}(k)$ instead of two equations for $E_{n}(k)$ and $E_{s}(k)$ separately.

## 3. Qualitative analysis of anisotropic two-dimensional energy rate equation

The presence of the mutual friction term in the RHS of (2.13) leads to decay of the function Ψ. As a result, E(k) decays even faster than in the K41 regime $E(q)\propto q^{-8/3}$, being very far from the thermodynamic equilibrium with E(k)∝k. In this regime, we can use a simpler algebraic closure for the energy flux (2.8a) instead of the differential closure (2.9a). This is equivalent to neglecting two last terms in the square brackets of (2.13). After division of the resulting equation by Ψ we get the simplified version of the energy rate (2.13)

3.1 $$(q\cdot \nabla_{q})\Psi (q)=-\frac{2\tilde{\Omega }q_{||}^{2}}{33C_{1}q^{2/3}(q_{||}^{2}+q_{\times }^{2})}.$$

Here, we took for simplicity $C(q)=C_{1}$.

For very small $q_{||}\ll q_{\times }$, in a zero-order approximation we can neglect the mutual friction term in the RHS of (3.1). Then $\Psi (q_{||},q_{⊥})≃\Psi (0.0)=\text{const}$. Note that $\Psi (q_{||},q_{⊥})$ is even function of $q_{||}$ and therefore has an extremum (presumably maximum) for $q_{||}=0$. This allows us to hope that $\Psi (q_{||},q_{⊥})$ can be roughly factorized as $\Psi (0,q_{⊥})\equiv \Psi_{||}(0)\Psi_{⊥}(q_{⊥})$ with $\Psi_{||}(0)=1$. In a more extended region, say, up to $q_{||}≲q_{\times }$, the mutual friction term becomes important and $\Psi_{||}(q_{||})$ decays fast with increasing $q_{||}$. As we show below, a significant (or complete) decay of $E(q_{||},q_{⊥})$ takes place in a narrow, compared to $q_{⊥}$, range of $q_{||}$. Therefore, in this case, we can interpret this phenomenon as a one-dimensional problem along $q_{||}$, in which $q_{⊥}$ and $\Psi_{⊥}(q_{⊥})$ can be considered as parameters. From the formal viewpoint, it means that we can accept (as a reasonable approximation) a factorization

3.2 $$\Psi (q_{||},q_{⊥})\approx \Psi_{||}(q_{||})\Psi_{⊥}(q_{⊥}),$$

neglect $q_{⊥}$-derivative and approximate q as $q_{⊥}$. All these simplify (3.1) as follows:

3.3 $$\frac{\text{d}\Psi_{||}(q_{||})}{\text{d}q_{||}}=-\frac{2\tilde{\Omega }q_{||}}{33C_{1}\Psi_{⊥}(q_{⊥})q_{⊥}^{2/3}(q_{||}^{2}+q_{\times }^{2})}.$$

To specify the boundary conditions, we introduce some $q_{*}$ in the beginning of the inertial interval (not necessarily equal to unity). Then, the solution of (3.3) with $\Psi_{||}(q_{*})=1$ is

3.4 $$\Psi_{||}(q_{||})=1-\frac{2\tilde{\Omega }\ln[(q_{\times }^{2}+q_{||}^{2})/(q_{*}^{2}+q_{\times }^{2})]}{33C_{1}\Psi_{⊥}(q_{⊥})q_{⊥}^{2/3}}.$$

We see that both $\Psi_{||}(q_{||})$ and $E(q)\propto \Psi_{||}(q_{||})$ vanish for some $q_{||}=q_{\text{cr}}$, for which $2\tilde{\Omega }\ln[1+{\left(q_{\text{cr}}/q_{\times }\right)}^{2}]=33C_{1}\Psi_{⊥}(q_{⊥})q_{⊥}^{2/3}$ and the RHS of (3.4) vanishes. This regime corresponds to so-called ‘super-critical regime’, first predicted in [46], studied in more details in [47] and numerically discovered in ${\;}^{3}\text{He}$ in [37]. It was shown that the super-critical regime appears for small $q_{\times }$ and very large $\tilde{\Omega }$. In this range of parameters, the mutual friction dominates over step-by-step cascade and the energy transfer loses its locality. This means that the energy flows directly from the small-wavenumber range into all larger q and is dissipated by the mutual friction at the same q. In this regime, the simple algebraic closure (2.8a) and its differential self-consistent improvement (2.9a) and (2.9b) become invalid even qualitatively and should be replaced, for example, by the non-local closure, suggested in [37]. DNS of superfluid turbulence in ${\;}^{3}\text{He}$, [37] and in ^4^He [14] shows that the energy spectrum in the super-critical regime remains scale-invariant but with the exponent m>4 (up to m≃10).

Probably, the most straightforward way to understand the behaviour of $\Psi_{⊥}(q_{⊥})$ is to return back to (2.10) and to integrate it over $k_{||}$ for fixed $k_{⊥}$. Then, the flux term in $k_{||}$ direction $\propto \partial [\ldots ]/\partial k_{||}$, responsible for the energy redistribution over $k_{||}$ vanishes and we get the rate equation for ${\;}^{⊥}E(k_{⊥})\equiv \int E(k_{||},k_{⊥})dk_{||}$

3.5*a* ∂ ⊥​E(k⊥,t)∂t−ddk⊥∫{…}dk⊥=−ωdis ⊥E(k⊥,t),

with the same expression in {…} as in (2.10). The choice of the effective frequency $\omega_{\text{dis}}$, responsible for the dissipation by mutual friction of the energy ${\;}^{⊥}E(k_{⊥},t)$ in the RHS of (3.5a), is very delicate. If we assume that the loss of the energy ${\;}^{⊥}E(k_{⊥},t)$ at some given $k_{⊥}$ is due to the mutual friction at the same $k_{⊥}$ and all $k_{||}$, then

3.5*b* $$\omega_{\text{dis}}={\tilde{\omega }}_{\text{dis}},\;{\tilde{\omega }}_{\text{dis}}\equiv \frac{\tilde{\Omega }\int \Psi_{||}^{2}(q_{||})q_{||}^{2}\;\text{d}q_{||}/\left(q_{||}^{2}+q_{\times }^{2}\right)}{\int \Psi_{||}^{2}(q_{||})\text{d}q_{||}}.$$

However, the main part of the energy ${\;}^{⊥}E(k_{⊥},t)$ is localized in the range of relatively small $k_{||}$ and the energy outflux from this region is suppressed in our model by the symmetry, because $\nabla_{k}\cdots =0$ for $k=\{0,k_{⊥}\}$ and small for small $k_{||}$. It is then reasonable to assume that $0.5<\omega_{\text{dis}}/{\tilde{\omega }}_{\text{dis}}<1$. In its turn, the ratio ${\tilde{\omega }}_{\text{dis}}/\tilde{\Omega }$ in the range of parameters (2.14) is close to unity. Therefore, considering $\omega_{\text{dis}}$ as a phenomenological parameter, we expect that $0.5<(\omega_{\text{dis}}/\tilde{\Omega })<1$.

Analysing equation (3.5) in the same manner as we did for (2.10), we arrive at the following equations for $\Psi_{⊥}(q_{⊥})$, similar to (3.3) for $\Psi_{||}(q_{||})$:

3.6*a* $$\frac{\text{d}\Psi_{⊥}(q_{⊥})}{\text{d}q_{⊥}}=-\frac{2\omega_{\text{dis}}}{33C_{1}q_{⊥}^{5/3}}.$$

Its solution with the boundary condition $\Psi_{⊥}(q_{*})=1$ is

3.6*b* $$\Psi_{⊥}(q_{⊥})=1-\frac{4\omega_{\text{dis}}(q_{*}^{-2/3}-q_{⊥}^{-2/3})}{99C_{1}}.$$

This equation, together with equations (2.11), (3.2) and (3.4), results in the semi-quantitative representation of the anisotropic two-dimensional energy spectrum of the unbounded counterflow turbulence with the axial symmetry:

3.7 $$E(q_{||},q_{⊥})≃\frac{E(q_{*})}{q^{8/3}}[1-\frac{2\tilde{\Omega }\ln[(q_{*}^{2}+q_{||}^{2})/(q_{\times }^{2}+q_{||}^{2})]}{33C_{1}\Psi_{⊥}(q_{⊥})q_{⊥}^{2/3}}{]}^{2}[1-\frac{4\omega_{\text{dis}}(q_{*}^{-2/3}-q_{⊥}^{-2/3})}{99C_{1}}{]}^{2}.$$

The explicit form (3.7) for the anisotropic energy spectra of counterflow turbulence is the main analytical result of our paper.

**Figure 1.**
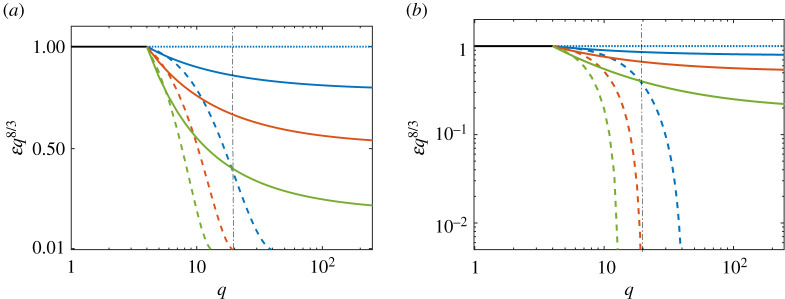
The K41-compensated spectra in direction of the counterflow (3.4), $q_{||}^{8/3}E(k_{||},0)=\Psi_{||}^{2}(q_{||})$ (dashed lines) and in the orthogonal direction, (3.6b), $q_{⊥}^{8/3}E(0,k_{⊥})=\Psi_{⊥}^{2}(q_{⊥})$ (solid lines). The parameters of the spectra $q_{\times }=20$, $\omega_{\text{dis}}=0.7\tilde{\Omega }$ and $q_{*}=4$. Three sets of lines from top to bottom correspond to $\tilde{\Omega }=2$ (blue lines), $\tilde{\Omega }=5$ (red lines) and $\tilde{\Omega }=10$ (green lines). Note the log-linear scales in (*a*) and the log-log scales in (*b*). Vertical black dot-dashed line denotes the $q_{\times }=20$. (Online version in colour.)

To explore the form of the two-dimensional-energy spectrum (3.7), we plot in figure 1 the cross-sections of the K41-compensated spectra in direction of the counterflow, $k_{||}^{8/3}E(k_{||},0)=\Psi_{||}^{2}(q_{||})$ ((3.4), dashed lines) and in the orthogonal direction $k_{⊥}^{8/3}E(0,k_{⊥})=\Psi_{⊥}^{2}(q_{⊥})$ ((3.6b), solid lines). The log-linear scales in figure 1*a* expose the details of $k_{⊥}^{8/3}E(0,k_{⊥})$, while the log-logs scale in figure 1*b* emphasize the strongly suppressed $k_{||}^{8/3}E(k_{||},0)$. We see that the spectra in the counterflow direction experience fast decay and sharp cut-off, corresponding to the super-critical regime in the approximation of the algebraic closure (2.8). On the other hand, the spectra in the orthogonal direction decay much slower, corresponding to the so-called ‘sub-critical regime’ [37,46,47] with the local (step-by-step cascade) energy transfer over scales. It consists of two K41 scaling laws: in the range of small q it has the energy flux $\varepsilon_{0}$ equal to the rate of the energy pumping, while for large q it has smaller energy flux $\varepsilon_{\infty }<\varepsilon_{0}$. The difference $\varepsilon_{0}-\varepsilon_{\infty }$ is dissipated on the way to large q due to mutual friction. At larger q, the dissipation by mutual friction is no longer efficient because scale-independent large-q asymptotic of the mutual friction frequency $\tilde{\Omega }$ becomes finally smaller than the K41 energy transfer frequency $\gamma (q)≃\varepsilon_{\infty }^{2/3}q^{2/3}$. A similar effect of vanishing of the mutual friction effect at small scales was originally observed in an isotropic system in [46].

We conclude that from the viewpoint of the qualitative analysis of the energy rate (2.13), the energy spectrum of counterflow turbulence has a pancake form around the counterflow direction $q_{||}$. It is strongly confined in the $q_{||}$ direction due to the special anisotropic form of the mutual friction force, effective only for $k_{||}\neq 0$. In the next section, we consider the numerical solution of the model (2.13) and compare the results with the qualitative predictions.

## 4. Numerical solution of energy rate equation and discussion

The equation (2.13) (with the replacement q→k) was solved numerically as a time evolution on the $500^{2}$-grid with the self-consistent form of $C_{1}(k)$ given by (2.12). We used the initial condition Ψ(k,0)=1 for all k. To reach the stationary solution, we added a forcing term with small amplitude $f_{0}=0.005$, acting in first four modes $k=\sqrt{k_{||}^{2}+k_{⊥}^{2}}\leq k_{*}=4$ and an artificial exponential dumping term, acting at the edges of the grid. After a short transient period, a steady-state solution for $\Psi (k_{||},k_{⊥})$ was obtained. We have verified that this solution is insensitive to the details of forcing and artificial dumping, as long as the stationary solution is reached.

**Figure 2.**
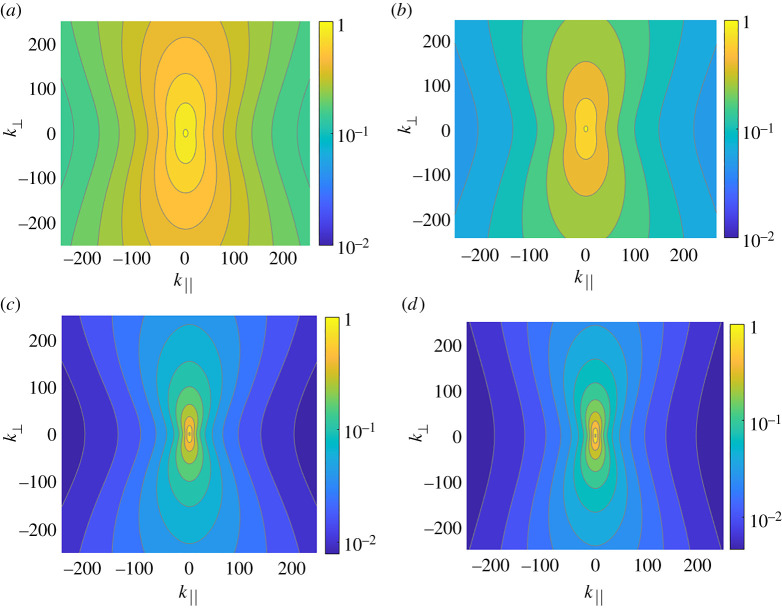
The K41-compensated two-dimensional energy spectra $k^{8/3}E(k)$. (*a*,*b*) The spectra are calculated for $k_{\times }=100$ and $\tilde{\Omega }=2,\;5,$ respectively. (*c*,*d*) The spectra are calculated for $k_{\times }=20$ and the same values of $\tilde{\Omega }$. Note logarithmic scale of the colour-bars. The contour levels are spaced by 0.1 in (*a*,*b*) and by 0.2 in (*c*,*d*). (Online version in colour.)

The contour plots of the two-dimensional energy spectra for several sets of parameters of the problems, $\tilde{\Omega }$ and $k_{\times }$, are shown in figure 2. The spectra are clearly confined along $k_{||}$, more strongly with increasing $\tilde{\Omega }$ and decreasing $k_{\times }$. Indeed, according to (2.12), larger $\tilde{\Omega }$ enhances the mutual friction, while smaller $q_{\times }$ increases the range in k-space where the mutual friction is important.

**Figure 3.**
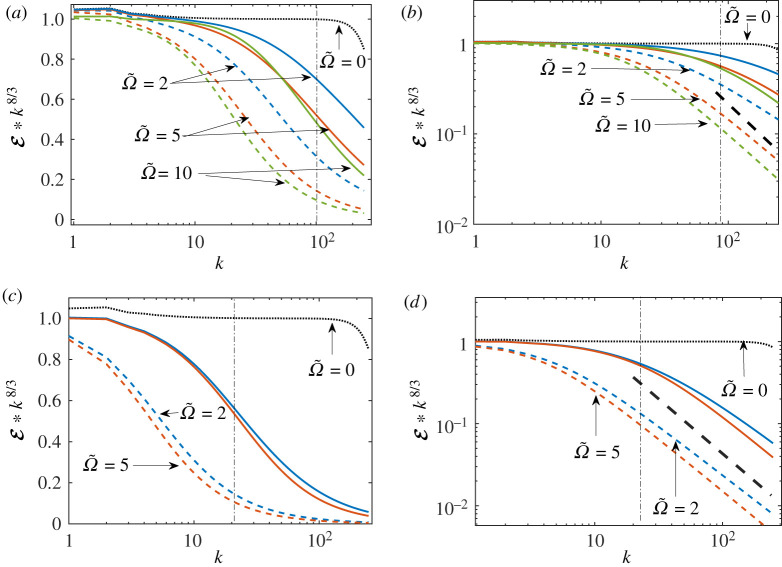
Numerical solution of (2.13). The K41-compensated spectra along $k_{||}^{8/3}E(k_{||},0)$ (dashed lines) and normal to the counterflow direction $k_{⊥}^{8/3}E(0,k_{⊥})$ (solid lines). The values of $\tilde{\Omega }$ are indicated in the figure. In (*a*,*b*) $k_{\times }=100$, in (*c*,*d*) $k_{\times }=20$. The reference case $\tilde{\Omega }=0$ (no mutual friction) is plotted in all panels by a black dotted line. Vertical dot-dashed lines denote the position of the crossover wavenumber $k_{\times }$. Black thick dashed lines in (*b*,*d*) denote $E\propto k^{-4}$ and serve to guide the eye only. Note the log-linear scales in (*a*,*b*) and the log-log scales in (*c*,*d*). (Online version in colour.)

The cross-sections of the two-dimensional compensated energy spectrum $k^{8/3}E(k)=|\Psi (k){|}^{2}$ are shown in figure 3*a*,*b* for $k_{\times }=100$ and in figure 3*c*,*d* for $k_{\times }=20$. The spectra $E(k_{||},0)$ along $k_{||}$, are shown by dashed lines and $E(0,k_{⊥})$ along $k_{⊥}$, by solid lines. Similar to figure 1, we plot the spectra both in the log-linear scales to emphasize the details of the orthogonal spectra, and in the more conventional log-log scales.

We see that spectra along the counterflow direction experience fast decay, while the energy cross-sections in the orthogonal direction decay much slower. For $k_{\times }=100$, the orthogonal spectra have some interval of the cascade-dominated range with near-K41 scaling that is shorter for larger $\tilde{\Omega }$. The spectra along $k_{||}$ do not have such an interval for these parameters. For $k>k_{\times }$, all spectra have similar power-law behaviour, which we discuss below. For $k_{\times }=20$, the spectra quickly saturate with increasing $\tilde{\Omega }$ and are almost completely in the mutual-friction-dominated range. However, due to self-consistent closure for the energy flux, the spectra do not become super-critical, as in the analytic solution.

An additional result of principle importance is the universality of the scaling exponent $x_{\text{cr}}=4$ of both longitudinal and transverse cross-sections of the energy spectra, $E(k_{||},0)\propto k_{||}^{-x_{\text{cr}}}$, $E(0,k_{⊥})\propto k_{⊥}^{-x_{\text{cr}}}$ shown in figure 3*b*,*d* by thick black dashed lines. The exponent $x_{\text{cr}}=4$ in two-dimensional energy spectra manifests itself in the so-called critical energy spectra, appearing in the regimes with strong enough mutual friction. The critical energy spectrum separates the sub-critical and the super-critical energy spectra with local and non-local energy transfer over scales [37], respectively. In the critical regime, the fraction of the energy loss due to mutual friction at each scale is about the fraction of the energy transferred down to smaller scales.

In our theory, the critical regime appears asymptotically in the range of parameters $q_{\times }$ and Ω, for which the dissipation by mutual friction becomes dominant. To compensate for the increasing loss of energy at each q, the energy flux (2.9) self-consistently adjusts the effective slope m of the two-dimensional spectra (2.9b) towards its critical value m=4, where the energy flux (2.9a) formally becomes infinite. Consequently, in our theory, the critical regime is reached for $k>k_{\times }$ in the wide range of the flow parameters. This conclusion is supported experimentally: in [33], the critical regime was observed in ^4^He counterflow for T=1.65,1.85,2.00 K and T=2.10 K. In this paper, the normal-fluid component of the counterflow is probed by ${\text{He}}_{2}^{*}$ molecular tracer-line tracking technique, allowing to measure one-dimensional plane-averaged energy spectrum ${\;}^{⊥}E(k_{⊥})$, connected to studied here two-dimensional-spectra $E(k_{||},k_{⊥})$ as follows:

4.1 $${\;}^{⊥}E(k_{⊥})=\int E(k_{||},k_{⊥})\text{d}k_{||}.$$

**Figure 4.**
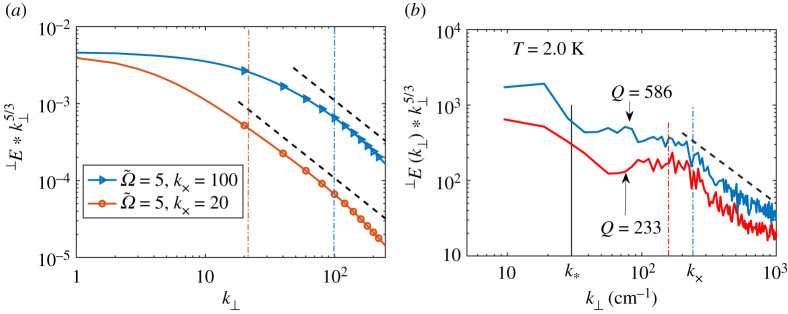
Comparison of the theoretical and experimental K41-compensated one-dimensional plane-averaged energy spectra ${\;}^{⊥}E(k_{⊥})k_{⊥}^{5/3}$. (*a*) The theoretical spectra (4.1), for $\tilde{\Omega }=5$ and two cross-over wavenumbers. (*b*) Experimental spectra measured by molecular-racer velocimetry [33], at T=2.0 K and two heat fluxes. The vertical dot-dashed lines of matching colours in both panels denote the position of the corresponding $k_{\times }$. Black dashed lines denote critical scaling ${\;}^{⊥}E(k_{⊥})\propto k_{⊥}^{-3}$. (Online version in colour.)

To compare our theory and experiment [33], we plotted in figure 4*a* the K41-compensated spectra $k_{⊥}^{5/3}{\;}^{⊥}E_{th}(k_{⊥})$, for $\tilde{\Omega }=5$ and two different $k_{\times }$. In figure 4*b,* we plotted the experimental spectra $k_{⊥}^{5/3}{\;}^{⊥}E_{\text{exp}}(k_{⊥})$, measured for T=2.00 K and two heat fluxes. In both theoretical and experimental spectra, we clearly see the two regimes with different apparent scalings: (i) in the region of small $k_{⊥}$ (roughly below and about $k_{\times }$)—non-universal apparent exponents that depend on the flow parameters and are close to the K41 scaling (almost horizontal lines for K41 compensated spectra) and (ii) the universal scaling with exponents, close to the critical value ${\tilde{x}}_{\text{cr}}=3$ for $k_{⊥}>k_{\times }$. Note that one-dimensional exponents differ by unity from their two-dimensional counterparts, e.g. in one dimension, the K41 scaling exponent ${\tilde{y}}_{K41}=5/3$ and ${\tilde{x}}_{\text{cr}}=3$, while in two dimensions, $y_{K41}=8/3$ and $x_{\text{cr}}=4$. We, therefore, infer that our theory reproduces two scaling ranges, previously found in laboratory experiments [33]: the cascade-dominated range in the range of small k with scaling ${\;}^{⊥}E(k_{⊥})\propto k_{⊥}^{-y}$, close to the K41 exponents $y≳\frac{5}{3}$ and the mutual-friction dominated range with the critical scaling ${\;}^{⊥}E(k_{⊥})\propto k_{⊥}^{-3}$.

## 5. Summary

We developed a theory of energy spectra in the thermally driven turbulent counterflow of superfluid ^4^He, which generalizes the L’vov–Pomyalov theory of counterflow turbulence [24] to the strongly anisotropic case. The theory is based on the gradually damped [30] coarse-grained equation (2.1) of the incompressible superfluid turbulence [14,36,37] and the novel anisotropic, self-consistent differential closure (2.9) for the vector of the turbulent energy flux ε(k). This closure combines the Kolmogorov-1941 dimensional reasoning [11], the Leigth-1968 differential form [44,45] to account for the possibility of the thermodynamic equilibrium and L’vov-Pomyalov-2018 self-consistent closure for the energy flux [24] that accounts for the dependence of the energy flux on the local slope of the energy spectrum in the window of its locality. In addition, the suggested closure prescribes the orientation of the vector of the energy flux ε(k) in the steepest-decent direction of three-dimensional turbulent energy spectra F(k) towards its thermodynamic equilibrium: $\varepsilon (k)||\nabla_{k}F(k)$.

Similar to previous theories [23,24], the important element of our theory is the anisotropic cross-correlation function (2.6) between the superfluid and normal-fluid velocity components. This function determines the rate of energy dissipation by the mutual friction in the final energy rate equation (2.10).

Detailed analysis of (2.10) leads to the analytic solution (3.7) for the energy spectrum that describes its strong suppression with respect to the classical fluid counterpart. The spectra are non-scale-invariant, and strongly depend on the temperature and the counterflow velocity in the wide range of these parameters. The resulting energy spectra of the normal-fluid and superfluid components are strongly confined in the direction of the counterflow velocity. This conclusion is supported by the numerical solution of the energy-rate (2.10) and by the direct numerical simulation of the coarse-grained equation (2.1) for the counterflow turbulence [29,30]. Our theory explains the critical scaling behaviour with the exponent ${\tilde{x}}_{\text{cr}}=3$ at $k>k_{\times }$, found in the experiment [33] that is insensitive to the flow parameters.

We, therefore, hope that the suggested theory captures the basic physics of the counterflow turbulence and describes the dependence of the anisotropic energy spectra on the main flow parameters.

## Data accessibility

This article has no additional data.
